# Lipidomics to Assess Omega 3 Bioactivity

**DOI:** 10.3390/jcm4091753

**Published:** 2015-09-07

**Authors:** Francesco Visioli

**Affiliations:** Department of Molecular Medicine, University of Padova, Via 8 Febbraio, 2-35122 Padova, Italy; E-Mail: francesco.visioli@unipd.it; Tel.: +390498276107; Fax: +3902700426106.

**Keywords:** lipidomics, omega 3 fatty acids, lipid analysis, lipidome, cardiovascular disease, clinical trials

## Abstract

How can we resolve the conflict between the strong epidemiological evidence pointing to the usefulness of fish—and, thus, omega 3—consumption with the debacle of supplementation trials? One potential explanation is that the null results obtained thus far are the consequences of ill-contrived investigations that do not allow us to conclude on the effects (or lack thereof) of omega 3 fatty acid supplementation. One potential solution is through the use of lipidomics, which should prove very useful to screen suitable patients and to correlate plasma (or red blood cells, or whole blood, or phospholipid) fatty acid profile with outcomes. This has never been done in omega 3 trials. The wise use of lipidomics should be essential part of future omega 3 trials and would help in untangling this current riddle.

## 1. Introduction

Fish consumption is directly associated with better cardiovascular prognosis [1]. From the first observations made in Greenland to the latest randomized controlled trials (RCT), long-chain omega 3 fatty acids, namely docosahexaenoic (DHA) and eicosapentaenoic (EPA) fatty acids have been held responsible for the health effects of fish [1]. One notable example is that of the omega 3 index, which is influenced by omega 3 consumption and is a strong predictor of cardiovascular risk [2]. Consequently, much research has been devoted to elucidating, *in vitro* and *in vivo*, the protective activities of EPA and DHA; indeed, the general perception is that supplementary intake of these fatty acids would protect from cardiovascular disease and other degenerative pathologies. Suggesting consumption of adequate amounts of omega 3 fatty acids actually makes biological sense; these fatty acids are important in that mammals cannot efficiently synthesize them and they are indispensable to a number of biological processes, especially those that involve excitable cells such as cardiomyocytes, neurons, retinal cells, *etc.* [1]. In addition, nearly every randomized clinical trial reported that omega 3 fatty acids positively modulate surrogate markers of cardiovascular disease and *in vitro* studies explored a wide range of mechanisms underlying the purported beneficial activities of EPA and DHA. The sad truth is that, when given as supplements, long chain omega 3 fatty acids are—very likely—ineffective in affording better health [3]. The big question is why [4]? What is wrong [5]? Indeed, this is probably the major current conundrum in pharma-nutrition research, with fish oils threatening to join the vitamin E, beta-carotene, resveratrol, *etc.* in the dustbin of promising nutritional therapies that failed to show benefit in RCTs [6,7,8].

## 2. Omega 3 Fatty Acids in Cardiovascular Therapy

How can we reconcile this apparent failure with strong epidemiological evidence pointing to the usefulness of fish—and, thus, omega 3—consumption? One potential explanation is that the null results obtained thus far are the consequences of ill-contrived trials that do not allow one to draw firm conclusions on the effects (or lack thereof) of omega 3 fatty acid supplementation [9].

In this respect, one paradigmatic example is that of folate in stroke prevention. In a well-designed trial recently published by Huo *et al.* [10], the highest risk of stroke and the greatest benefit of folic acid therapy were seen in patients with the lowest baseline folate levels. In addition, Huo *et al.* [10] suggest that individuals with the TT genotype may require a higher dosage of folic acid supplementation to overcome biologically-insufficient levels (as reflected in the relatively greater folate requirement for subjects with the TT genotype).

Unfortunately, neither basal plasma concentrations nor genotype/nutrigenomics issues have ever been taken into consideration in omega 3 supplementation studies [9]. The natural consequence is that the near totality of omega 3 trials could not really conclude that modulation of omega 3 status is ineffective. This situation is unheard of in classic drug research, where surrogate markers, e.g., blood pressure of cholesterol concentrations, are mandatorily assessed before and after interventions on these risk factors.

In this respect, the use of lipidomics in RCTs should prove very useful to screen patients first and to correlate plasma (or red blood cells, or whole blood, or phospholipid) fatty acid profile with outcomes.

## 3. Lipidomics

From a technical viewpoint, lipidomics can be conveniently classified into as two distinct approaches: A “shotgun”, *i.e.*, electrospray ionization coupled with single stage or tandem mass spectrometry approach and a liquid chromatography-mass spectrometry (LC-MS)-based one. The shotgun approach does not require separation prior to MS analyses. The shotgun approach is being considered as the best method for analysis of lipid extracts. Its major advantage over other methods is that mixtures of lipids are directly infused and sprayed into the mass spectrometer, therefore analyzing all the molecules collectively [11]. In this way, shotgun lipidomics, when applied to whole tissue lipid extracts, generates molecular ions without extensive fragmentation of the molecules. This type of lipidomics allows the identification and quantitation of the lipids in complex mixtures and provides helpful global information about cellular and tissue lipidomes. The procedures generate quasi-molecular ions in high yields. The major limitation of shotgun lipidomics is that the most abundant or most efficiently ionized molecules “quench” the signal of other ones, in turn causing ion suppression and hampering detection of lipids that are present in lower concentrations [12]. Therefore, intra-source separation is often employed to overcome this limitation. Mostly, the analytical setting of lipidomics involves a triple-quadrupole mass spectrometer with electrospray ionization (ESI, primarily negative mode) in selective reaction monitoring (SRM) or multiple reaction monitoring (MRM) to maximize sensitivity [12]. Whereas reversed-phase chromatography, *i.e.*, C18, is the first separation choice, hydrophilic interaction liquid chromatography (HILIC) is best for phospholipids, sphingolipids, and phosphatidylcholines [12]. In summary, the most appropriate methodological approach should be based on the goals of the analysis and, in the future, we might witness a combination of techniques that would match ease of use with accuracy.

When mass spectral equipment is available in clinical laboratories, lipidomics can be applied to clinical studies. Current bottlenecks include sample handling methods and data reduction and analysis of the enormous volume of data generated by contemporary mass spectrometric analysis. However, this rapidly evolving field can rapidly elucidate the lipidome in medical samples of small size. Of note, current techniques can identify thousands of distinct lipid molecular species, of which the most biologically important ones are fatty acyls, glycerolipids (neutral), glycerophospholipids (polar), sphingolipids, sterols, and prenols [13,14].

## 4. Lipidomics Applied to Fatty Acid Research

As mentioned, the lipidome provides useful information on the basal omega 3 status of patients or healthy volunteers and can greatly help resolve the current omega 3 conundrum in cardiovascular research/clinic. To date, lipidomics have been applied to observational and preclinical models of cardiovascular disease, including mice, hamsters, rabbits, and pigs, and nonhuman primates.

Example of observational research include the use of lipid profiling in diabetes mellitus [15,16], cardiovascular disease [17,18], metabolic disorders [19], dietary habits, and obesity [20], as well as in determining response to drug therapy (elegantly reviewed by Hinterwirth *et al.* [13]). These techniques can provides useful information on the lipid composition and profile of whole blood or of tissues e.g., the retina [21], atherosclerotic plaques [22], muscles [23], or adipose tissue [24].

In terms of cardiovascular disease and lipid profiling, some examples are worth our attention. The use of olive oil as a predominant source of fat has been consistently associated with better cardiovascular prognosis. Most researchers are still attributing the purported healthful properties of olive oil to its relatively high monounsaturated fatty acids (MUFAs) content [25]. The theory behind this is that MUFAs, namely oleic acid, favorably modifies the high-density lipoprotein/low-density lipoprotein (HDL/LDL) ratio and, therefore, afford cardiovascular protection [25]. Yet, accumulated evidence clearly indicates that MUFAs only mildly modify the HDL/LDL ratio, to an extent that should minimally influence cardiovascular risk [26]. Moreover, at least two lipidomic studies [27,28] show that the proportion of MUFAs is higher in red blood cells and whole blood, respectively, of myocardial infarct patients as compared with controls, confirming findings from the large dataset of Stegeman *et al.* [17]. In summary, the hypothesis that MUFAs are cardioprotective and that increasing their intake would benefit the cardiovascular system is still unproven.

Lipidomics have also been employed in pharmaceutical research [11], namely to assess the effects of selected drugs on hypertension [29] and cardiovascular disease, in particular when recombinant HDL [16] and statins [30] were employed.

Within the context of this paper, it is worth reiterating that lipidomics have never been applied to omega 3 intervention trials and that precisely this improper approach is leading to pallid conclusions on their therapeutic roles.

## 5. Examples of Lipidomics Applied to Omega 3 Research

**Figure 1:**
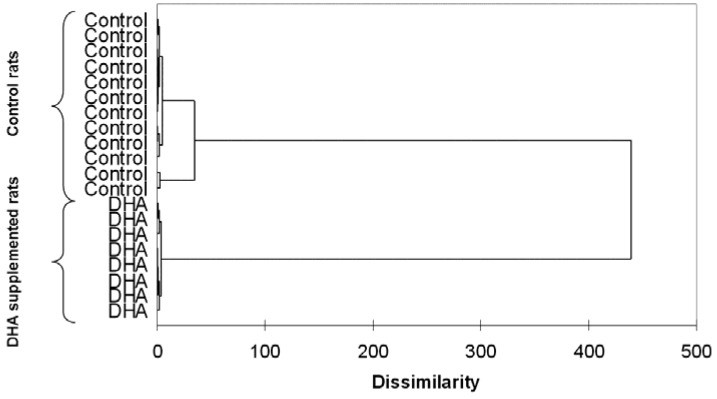
Example of lipidomics applied to omega 3 research: dendrogram resulting from the hierarchical cluster analysis of atrial rat cardiomyocytes isolated from control and docosahexaenoic (DHA)-treated rats. Unpublished data. Methodological details can be found in Lamaziere *et al.* [33].

As mentioned, the near totality of omega 3 trials did not include baseline or post-treatment evaluations of omega 3 status in patients of healthy volunteers. In rats, lipidomics have been successfully employed by Lamaziere and coworkers to assess the effects of omega 3 supplementation on hepatic lipogenesis [31] and brain composition [32]. Additionally, this technique allowed the identification of the *in vitro* concentrations to be employed in cardiac biochemistry studies [33]. It is worth noting that, even though rats are given identical feed, their tissutal omega 3 status varies greatly [31,32,33]. One can only speculate that these differences are enhanced in humans who consume varied diets. Additionally, omega 3 supplementation reduces such inter-individual variations (Figure 1) and renders the omega 3 composition of organs and tissues more homogeneous [31,32,33].

Another mechanistic application of lipidomics to omega 3 research concerns the differential distribution of DHA and EPA in the various lipid classes. As an example, the former mostly accumulates into phospholipids whereas the latter contributes to cholesterol esters [34]. Likely, the biological importance of phospholipids is higher than that of cholesterol esters because they originate from important lipid mediators; their compositional modulation by diet or supplement should result into more relevant biological actions.

Finally, we should not forget that the *in vivo* effects of omega 3 fatty acids likely depend on capsules’ formulation, *i.e.*, tryacylglycerols *vs.* ethyl esters *vs.* phospholipids [35] and on whether they are taken as supplements or as part of food items (either food as such [36] or functional food such as milk [37,38]). It is conceivable that omega 3 fatty acids’ bioavailability and subsequent distribution in the body might be different depending on the matrix [35]. This can be explored by proper lipidomic approaches.

In recent years, much investigation has been dedicated to bioactive small molecules that activate the resolution of acute inflammation [39]. These molecules are autacoids that include resolvins*,* protectins, and maresins, which are formed from EPA and DHA during acute inflammation. Interestingly, resolvins and protectins can currently be synthesized [40], which paves the way to their future use in therapy. Lipid-mediator lipidomic profiling of self-resolving exudates (after solid-phase extraction) allowed framing the time course of inflammation resolution and elucidating the roles played by these important omega 3-derived molecules [40]. Alas, the lipidomic methods that have been successfully employed to clarify the lipid mediator profiles of phagocytes and inflammatory exudates [41] have never been employed in omega 3 clinical trials, thus preventing drawing firm conclusions on their real contribution to the resolution of inflammation in humans.

## 6. Conclusions

While the assessment of relevant biomarkers, e.g., blood pressure, glycaemia, or cholesterol is habitual in pharmacological research, research in the omega 3 area has been hampered by the lack of baseline and post-treatment measurements of fatty acid levels. This prevents drawing solid conclusions on the true healthful role of these essential fatty acids. While it is tempting—based on cumulated evidence—to state that the omega 3 myth has been debunked, we need much more focused research before solid conclusions be reached and before we can educe evidence-based recommendations for or against using omega 3 supplements. This is even more relevant for cardiovascular patients who, as reviewed elsewhere [9], are treated with effective and potent drugs and might enjoy additional benefits from omega 3 supplementation only if their basal concentrations are low, *i.e.*, with an omega 3 index <4%.

The wise use of lipidomics should be essential part of future omega 3 trials and would help untangle this current riddle.
